# Supplementary material for: Relative Telomere Length Is Associated with the Risk of Development and Severity of the Course of Age-Related Macular Degeneration in the Russian Population
Source: Int J Mol Sci. 2023 Jul 12;24(14):11360. doi: 10.3390/ijms241411360 (PMC10379970; doi:10.3390/ijms241411360)
Supplement: Supplementary file 1 [file ijms-24-11360-s001.zip › ijms-2470575-supplementary.pdf]

Supplementary Table S1: Sensitivity and specificity analyses for the logistic regression analysis investigating the association of AMD using different adjustment variables.

**Model**	**OR (95% CI)**	**p-value**	**sensitivity**	**specificity**
Model 0<br>Unadjusted	**1.371 - 1.871**	**0.045**	-	-
Model 1 adjusted for sex	**1.529 (0.837 - 2.869)**<br>Sex - 0.864 (0.420 - 1.743)	0.173<br>Sex - 0.685	-	-
Model 2 adjusted for age and sex	**0.518 (0.063 - 4.188)**<br>Sex - 0.839 (0.406 - 1.699)<br>Age - 1.016 (0.987 - 1.046)	0.536<br>Sex - 0.628<br>Age - 0.288	0.071	0.958
Model 3 adjusted for age, sex and current smoking	**0.671 (0.072 - 6.281)**<br>Sex - 0.705 (0.279 - 1.685)<br>Age - 1.015 (0.986 - 1.045)<br>Smoking -0.693 (0.228 - 2.072)	0.725<br>Sex - 0.441<br>Age - 0.326<br>Smoking - 0.510	0.086	0.938
Model 4 adjusted for age, sex, current smoking, cardiovascular disease, hypertension, obesity and physical inactivity	**4.868 (0.340 - 73.800)**<br>Sex - 0.241 (0.078 - 0.685)<br>Age - 0.988 (0.954 - 1.023)<br>Smoking - 0.407 (0.112 - 1.448)<br>Cardiovascular disease - 3.506 (1.506 - 8.644)<br>Hypertension - 0.453 (0.124 - 1.578)<br>Obesity - 3.003 (1.108 - 8.77)<br>Physical inactivity - 6.694 (2.705 - 18.331)	**0.246**<br>Sex - 0.009*<br>Age - 0.507<br>Smoking - 0.166<br>Cardiovascular disease - 0.005 *<br>Hypertension - 0.218<br>Obesity - 0.036 *<br>Physical inactivity - **0.0001***	0.757	0.666
Model 5 adjusted for sex, cardiovascular disease and physical inactivity	**0.929 (0.467 - 1.854)**<br>Sex - 0.428 (0.183 - 0.958)<br>Cardiovascular disease - 2.328 (1.131 - 4.889)<br>Physical inactivity - 7.181 (3.123 - 18.310)	0.833<br>Sex - **0.043***<br>Cardiovascular disease - **0.023***<br>Physical inactivity - **<0,00001 ***	0.871	0.583

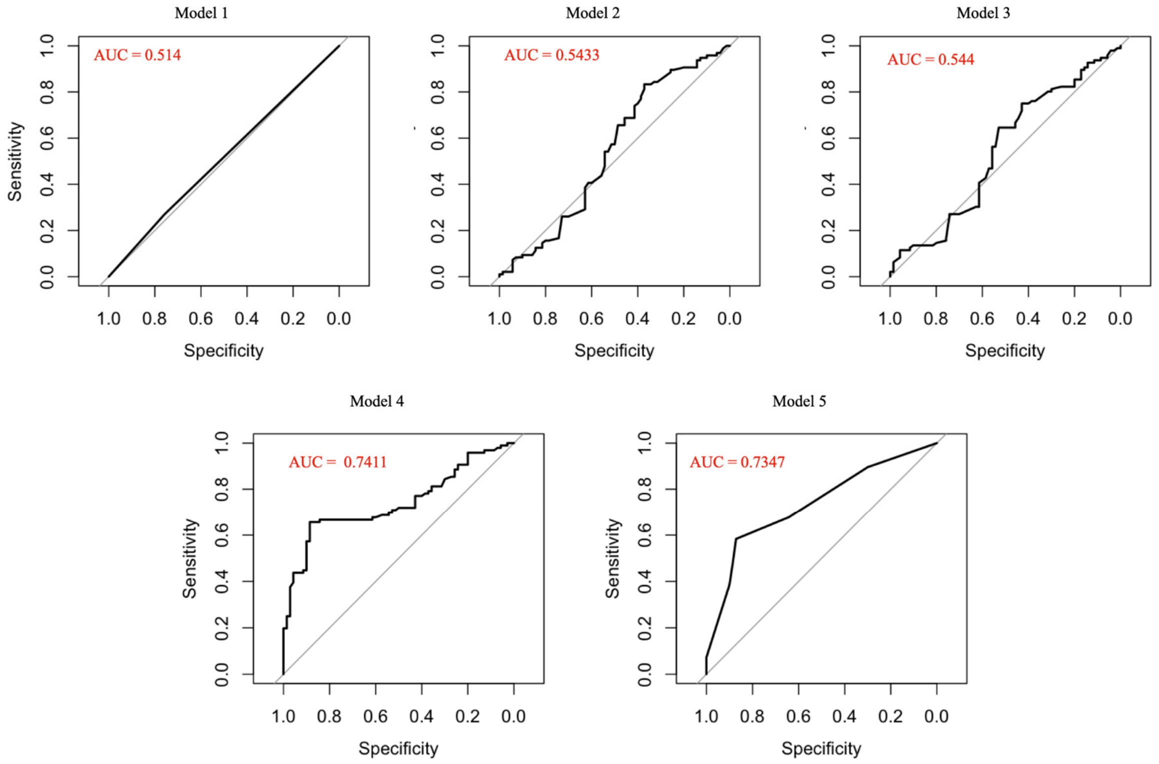

Supplementary Figure S1. ROC curves for Logistic Regression Models without RTL data. Model 1 receiver operating characteristic (ROC) curve adjusted for sex. Model 2 ROC curve adjusted for age and sex. Model 3 ROC curve adjusted for age, sex and current smoking. Model 4 ROC curve adjusted for age, sex, current smoking, cardiovascular disease, hypertension, obesity and physical inactivity. Model 5 ROC curve adjusted for sex, cardiovascular disease and physical inactivity. Area under the curve (AUC) values are the probability that a randomly selected 'case' (here, a AMD) is ranked as being at greater risk of being a 'case' than a randomly selected control of the same covariates.

Supplementary Table S2: Sensitivity and specificity analyses for the logistic regression analysis investigating the association of RTL and AMD using different adjustment variables.

RTL	All, n = 166		Women, n = 119		Men, n = 47	
	**sensitivity**	**specificity**	**sensitivity**	**specificity**	**sensitivity**	**specificity**
Model 1 adjusted for sex	0.829	0.927	-	-	-	-
Model 2 adjusted for age and sex	0.814	0.917	0.830	0.914	0.824	0.808
Model 3 adjusted for age, sex and current smoking	0.814	0.917	0.8301887	0.914	0.765	0.885
Model 4 adjusted for sex, cardiovascular disease and physical inactivity	0.843	0.938	0.8235294	0.923	0.882	0.923
